# The influence of nest site characteristics on Asian tits (*Parus cinereus*) defense behavior

**DOI:** 10.7717/peerj.21608

**Published:** 2026-07-22

**Authors:** Mingju E, Chao Shen, Ziyang Hu, Yingxue Cao, Li Liu, Xudong Li

**Affiliations:** 1 School of Life Sciences, Changchun Normal University, Changchun, China; 2 College of Life Science, Shenyang Normal University, Shenyang, China; 3 School of Ecology and Environment, Baotou Teachers’ College, Baotou, China

**Keywords:** Nest site selection, Cavity-nesting bird, Nest defense behavior, Asian tits

## Abstract

Birds can assess risk posed by predators in their environment and adjust their anti-predation strategies accordingly. However, the impact of nest site characteristics on nest defense behaviors has not been thoroughly studied. This study aimed to investigate the relationship between nest defense behaviors and nest site characteristics in Asian tits (*Parus cinereus*) when confronted with Common chipmunks (*Tamias sibiricus*, ground predator) and Eurasian sparrowhawks (*Accipiter nisus*, aerial predator). Generalized linear mixed-model analyses revealed that predator type significantly influenced both aggressive and vigilance behaviors, with Asian tits less likely to exhibit these two behaviors toward Eurasian sparrowhawks than toward Common chipmunks. Nest-site shrub height negatively predicted the probability of vigilance behavior, whereas no measured nest-site characteristics influenced aggressive behavior. Neither predator type nor nest-site features significantly affected stationary observation or threatening behaviors. These findings demonstrate that Asian tits adopt predator-specific defensive strategies: they adjust vigilance behavior s according to local shrub height while maintaining stationary observation and threatening behaviors relatively invariant, supporting their capacity for dynamic risk assessment in heterogeneous predation environments.

## Introduction

Adaptive nest site selection is an anti-predation strategy used in predation risk scenarios. Birds collect information about predators in the vicinity of the nest by sight, sound, and smell, and they can adjust nest site selection according to the perceived risk (Lima, 2009). Although birds try to reduce the risk of predation around them during nest site selection, predation is an unpredictable event that remains one of the primary factors that impact on their reproductive success (Chalfoun & Martin, 2010). Some studies found that birds can assess the level of risk in their environment and adjust the strength of their nest defense behavior based on certain nest site characteristics (Kleindorfer, Fessl & Hoi, 2005; Mérő & Žuljević, 2017; Yin et al., 2023). Therefore, the intensity of their defense response against predators may depend on the type of predator that approaches the nest.

Nest concealment is one of the main factors affecting nest security and is usually influenced by the density and height of surrounding vegetation (Rohrbaugh Jr & Yahner, 1997; Berl et al., 2014; Cantor et al., 2019). Specifically, the vegetation around nest boxes can shield cavity entrances and thus reduce the likelihood of nest discovery and predation; this, in turn, enhances breeding performance (Fisher & Wiebe, 2006; Bellamy et al., 2018). Ricklefs (1977) reported that the strength of nest defense behavior in tropical finch birds was negatively correlated with nest concealment. Strong nest defense behavior might cause birds to expose otherwise hidden nest sites (Ricklefs, 1977). Therefore, for birds with hidden nests, avoiding intense nest defense behavior is optimal for conserving energy as well as avoiding exposure to the nest site.

Nest accessibility is also one characteristic that influences the strength of nest defense behavior in birds. If a nest is difficult to approach by predators, then it may effectively reduce the input of nest defense (Yin et al., 2023). In addition, choosing a higher nest position can reduce the risk of predation (Matysioková & Remeš, 2024), as nests at lower sites are more vulnerable to attacks by ground predators and damage by humans (Kleindorfer, Fessl & Hoi, 2005). For example, reed warblers (*Acrocephalus scirpaceus*) with lower nesting positions showed a higher intensity of nest defense behavior against ground predators, whereas reed warblers with higher nesting positions showed a higher intensity of nest defense behavior against aerial predators (Kleindorfer, Fessl & Hoi, 2005). There is growing evidence that birds can distinguish between different types of nest intruders and adapt their nest defense behavior based on the threat of intruders and distance from the nest (dynamic risk assessment hypothesis) (Kleindorfer, Fessl & Hoi, 2005; Sam & Fuchs, 2010).

The Asian tit (*Parus cinereus*) is a secondary cavity-nesting species that does not excavate its own cavities. Formerly classified as the Japanese tit (*Parus minor*), this bird is now recognized as a subspecies (*Parus cinereus minor*) of the Asian tit (AviList Core Team, 2025). As non-migratory residents, these tits occupy breeding areas year-round, allowing sufficient time to evaluate nest-site quality. Two main predators were present at our study site: Common chipmunks (*Tamias sibiricus*, ground predator) and Eurasian sparrowhawk (*Accipiter nisus*, aerial predator). We chose Asian tits for the study species and observed their behavioral responses to predators by placing taxidermic specimens of Common chipmunks and Eurasian sparrowhawks above the nest box. We measured four nest site characteristics: canopy cover, shrub height and density around the nest, and nest height. These characteristics were selected for their effect on nest visual concealment and predation risk, and thus affect nest survival. Furthermore, we investigated relationships between these four nest site characteristics and parental nest defense intensity. Our objectives were (1) to determine whether the nest-defense responses of Asian tits were affected by predator type, and (2) to examine whether preferred nest-site characteristics of Asian tits were associated with the intensity of their nest-defense responses.

## Materials & Methods

Experiments were carried out in the Zuojia Nature Reserve (44°1′–45°0′N, 126°0′–126°8′E) in Jilin, northeast China. Since 2009, we placed about 400 artificial nest boxes for secondary cavity-nesting birds in the sample plots and monitored the boxes for occupancy by Asian tits every 5 days while recording breeding parameters. When the eggs were close to hatching, we inspected the nest boxes daily to record the exact hatching date. Nest height (cm) was measured using a tape measure as the vertical distance from the ground to the bottom of the nest-box entrance. Canopy cover above each nest box (0–100%) was estimated by vertical observation from four cardinal directions 1-m away from the nest tree. Shrub height (cm) around each nest box was quantified as the mean height of 10 randomly selected shrubs within a 1-m radius around the nest tree. Shrub density within a 1-m radius of each nest box (0–100%) was assessed from four cardinal directions around the nest tree.

From April to May 2019–2021, we conducted predator-simulation experiments during the chick-rearing period when the nestlings were 5–10 days old. We used taxiderm specimens of Common chipmunks (nest predator) and Eurasian sparrowhawk (adult predator) as predator models. In total, we tested 12 nests in 2019, 18 in 2020, and 15 in 2021.

Experiments were conducted on clear, windless days from 8:00 a.m. to 5:00 p.m. Once it was confirmed that parent birds were not moving around the nest, the specimens were quickly placed on the lid of the nest box. The experiment took place at a distance of 10 to 15-m from the nest. When the parent birds approached within 10-m of the nest, the experiment was timed, and their response behavior was recorded in detail for 5 min. The recorded response behavior included: (1) stationary observation (yes or no) where the parent returned to the nesting area, stood still and observed; (2) vigilance behavior (yes or no) where the parent returned to the nesting area and exhibited jumping accompanied by wing flashing; (3) threatening behavior (yes or no) where the parent approached the intruder closely, unfurled its tail feathers, beat its wings heavily, and slowly swung its body while hissing-a behavior usually accompanied by hovering, and (4) aggressive behavior (yes or no) where the parent bird directly charged at the intruder and occasionally made physical contact. Direct attacks by Asian tits during the experiment could cause serious damage to the specimens; therefore, the experiment was terminated if the birds physically attacked the specimens more than five times (Ippi et al., 2013). Since two predator species were placed in each nest, the order of specimens was randomized, and the interval between experiments was at least one hour. Additionally, specimens used in the present study were in their natural state, and two or more specimens were used for each species. If both parent birds did not return within 30 min after placing the specimens, the experiment was terminated and repeated after at least one hour. Different specimens from each nest were used to complete the experiment in one day to avoid the influence of weather and other factors. Only one complete round of behavioral testing (including both predator species) was carried out per nest.

### Ethical approval statement

This study was reviewed and approved by the Ethics Committee of Changchun Normal University (Approval No. 201908). This field survey was approved by Zuojia Nature Reserve and Forestry Bureau of Jilin Province (Approval No.: (2006) 178). All field procedures involving birds were conducted in accordance with the ethical standards of the institutional research committee and complied with relevant national wildlife protection regulations and international guidelines for field research on birds. Efforts were made to minimize disturbance to the birds and their habitats. No birds were captured, harmed, or subjected to invasive procedures during the study.

### Data analysis

Data were analyzed using R software version 4.6.0 (http://www.r-project.org). Prior to model construction, we calculated the variance inflation factor (VIF) to assess multicollinearity among continuous nest-site variables; all VIF values were below 1.2, indicating no severe multicollinearity. We evaluated the effects of predator type and nest site characteristics (canopy cover, shrub height, shrub density, and nest height) on four distinct nest defense behaviors of Asian tits (stationary observation, vigilance, threatening, and aggressive behaviors), each of which was treated as a binary response variable (yes/no). For each behavior, we fitted a generalized linear mixed model (GLMM) with a binomial error distribution and logit link function using the lme4 package (Olah et al., 2014). The full model included predator type, canopy cover, shrub height, shrub density, and nest height as fixed effects, with year and nest box ID as random intercepts to account for inter-annual variation and repeated measurements within the same nest. Continuous nest-site variables were standardized (mean = 0, SD = 1) prior to analysis to facilitate model convergence and comparison of effect sizes. We assessed the significance of fixed effects using Type II Wald chi-square tests with the car package. The threshold for statistical significance was *P* < 0.05. All graphical visualizations were generated with the ggplot2 package (Wickham, 2016).

## Results

**Figure 1:**
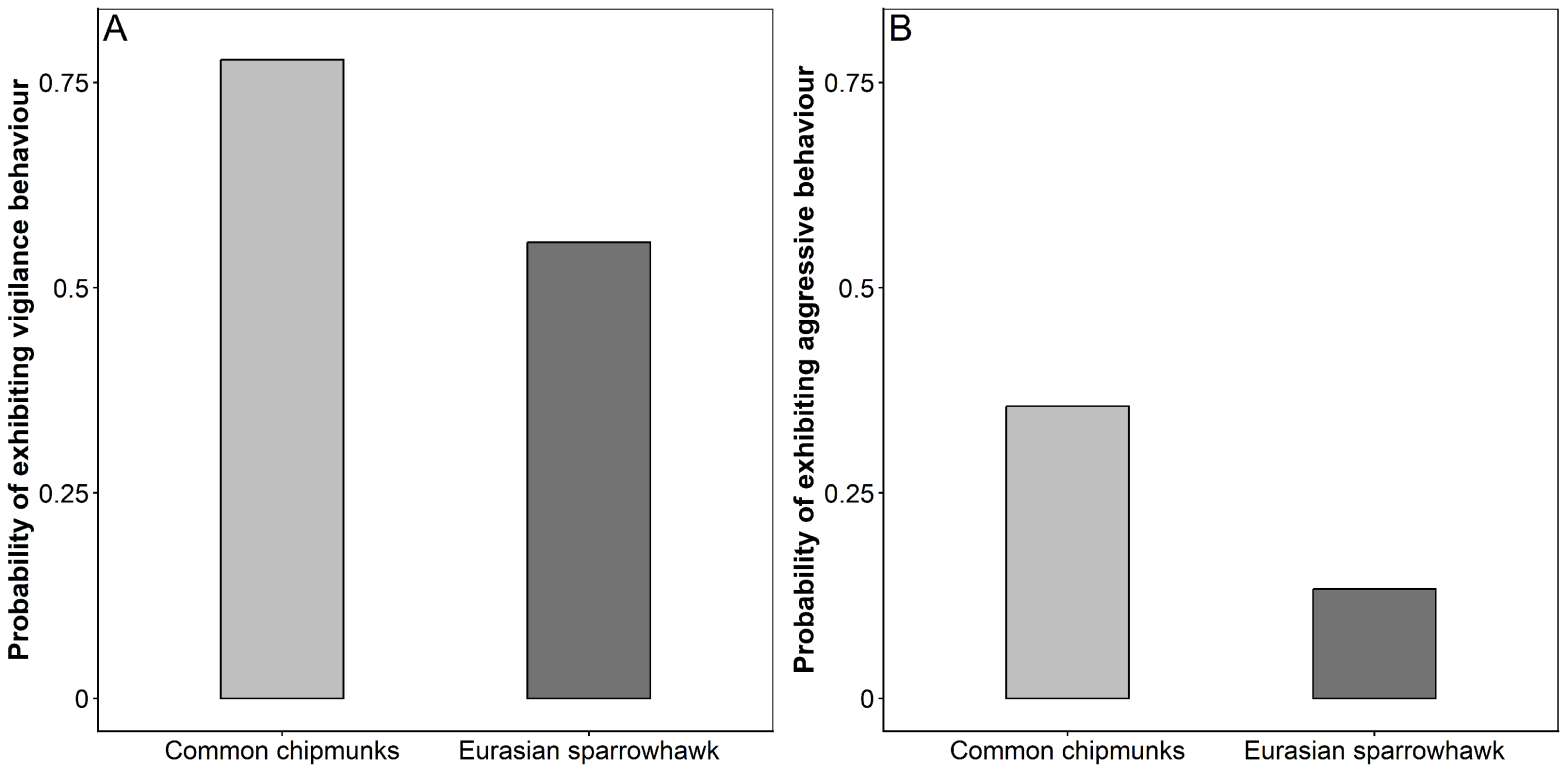
Probability of vigilance and aggressive behaviours displayed by Asian tits when exposed to two predator stimuli (common chipmunks and Eurasian sparrowhawks).

**Figure 2:**
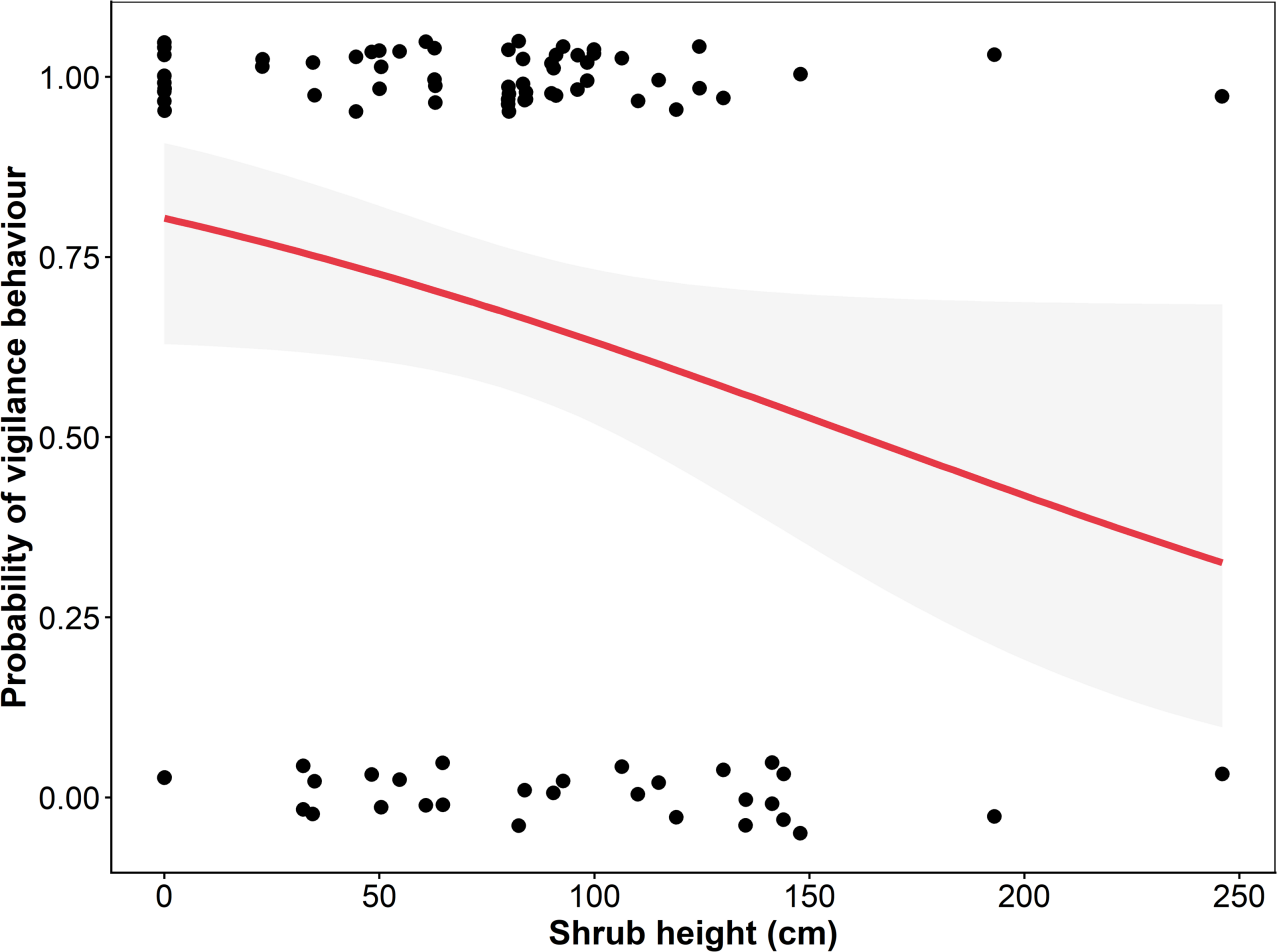
Relationship between shrub height around nests and the probability of vigilance behaviour in Asian tits.

When confronted with different predators, Asian tits exhibited aggressive behavior, and predator type significantly influenced whether they displayed a particular behavior (GLMM: *χ*^2^ = 5.81, *df* = 1, *P* = 0.016). The probability of aggressive behavior in Asian tits was lower when confronted with Eurasian sparrowhawks than with common chipmunks (Fig. 1). However, canopy cover, shrub height, shrub density, and nest height did not significantly affect the aggressive behavior (all *P* ≥ 0.176). When exposed to different predators, Asian tits also exhibited vigilance behavior, with both predator type and shrub height significantly affecting the likelihood of behavior (predator type: *χ*^2^ = 5.11, *df* = 1, *P* = 0.02; shrub height: *χ*^2^ = 3.94, *df* = 1, *P* = 0.047). The probability of vigilance behavior was lower when Asian tits were confronted with Eurasian sparrowhawks compared to common chipmunks (Fig. 1). Additionally, the probability of Asian tits exhibiting vigilance behavior decreased significantly with increasing shrub height around nests (Fig. 2). Canopy cover, shrub density, and nest height did not significantly influence whether Asian tits exhibited vigilance behavior (all *P* ≥ 0.436). Furthermore, predator type, nest height, canopy cover, shrub height, and shrub density did not significantly affect stationary observation and threatening behavior (all *P* ≥ 0.298).

## Discussion

When confronted with different predator stimuli, Asian tits exhibited distinctive aggressive and vigilance responses that were primarily influenced by predator identity, whereas nest site vegetation characteristics only affected vigilance behavior, but not aggression. Our results showed that Asian tits were less likely to display both aggressive and vigilant behavior toward Eurasian sparrowhawks compared to Common chipmunks. Eurasian sparrowhawks are large aerial predators that pose a direct threat to adult parent birds, but they cannot access tree cavity nests to harm nestlings. In contrast, common chipmunks are small terrestrial predators that specifically target eggs and nestlings within cavities. This fundamental difference in predation risk likely explains the reduced defensive behaviors toward Eurasian sparrowhawks. Parent tits prioritize self-survival when confronted with Eurasian sparrowhawks, whereas they invest more in active anti-predator behavior against Common chipmunks to protect their offspring. This pattern aligns with life-history trade-offs between parental survival and reproductive success (Montgomerie & Weatherhead, 1988; Ippi et al., 2013).

Notably, shrub height around nests significantly and negatively predicted the probability of vigilance behavior across predator treatments, whereas no nest site variables influenced aggressive behavior. Taller shrubs create denser vertical vegetation surrounding nest boxes, providing Asian tits with visual concealment and refuge from aerial predators such as sparrowhawks. Dense shrub cover can reduce predation risk by decreasing predator search efficiency while at the same time also reducing the need for continuous vigilance monitoring (Montgomerie & Weatherhead, 1988; Gómez-Serrano & López-López, 2014). In contrast, aggressive behavior is a high-cost, direct confrontational strategy that depends more on the perceived immediate predation threat rather than on fine-scale vegetation structure. This may explain why canopy cover, shrub height, shrub density, and nest height showed no significant effects on aggression. Vegetation characteristics may modulate risk perception for low-cost vigilance behavior but have limited capacity to influence high-risk aggressive responses.

We found that neither nest site characteristics (canopy cover, shrub height, shrub density, nest height) nor predator type significantly affected the stationary observation and threatening behavior of Asian tits (all *P* ≥ 0.298). This finding indicates that these two defensive behaviors are relatively stable and less responsive to variations in both predator identity and microhabitat conditions. Previous studies from our research group (Yin et al., 2023) documented that this same species (referred to as Japanese tits in their work) actively exhibited threatening and aggressive behavior against Common chipmunks. Regarding stationary observation, this lack of responsiveness may be attributed to its functional role as a basic, low-cost risk assessment behavior. Asian tits maintain a consistent level of stationary observation regardless of predator type or nest site features, as it serves as a foundational strategy to monitor potential threats without incurring high energy costs.

Regarding threatening behavior, the lack of observable effects from both predator type and nest site characteristics reflect a balance of opposing ecological pressures. Dense vegetation can simultaneously conceal nests from chipmunks and provide climbing pathways for these ground predators to access cavities (Martin, 1988), thus creating conflicting selective forces that may negate measurable impacts of shrub density, canopy cover, and shrub height on threatening behavior. Similarly, the difference in predation risk posed by sparrowhawks and chipmunks may not elicit divergent threatening responses, because threatening behavior is a relatively mild defensive strategy that does not escalate to direct confrontation, making it less sensitive to predator type. Additionally, Asian tits often select nest sites with moderate vegetation cover to balance the risk of predator detection and access (Li et al., 2023), which may further homogenize their threatening and stationary observation responses regardless of minor variations in nest site features or predator identity.

Nest height also showed no influence on any defensive behavior in our study, likely because all nest boxes were artificially installed at similar heights, to eliminate the natural height variation that typically affects the detection of climbing predators (Dyson, Slattery & Fedy, 2019).

## Conclusions

This study has demonstrated that Asian tits exhibit predator-specific nest-defense adjustments consistent with the dynamic risk-assessment hypothesis (Kleindorfer, Fessl & Hoi, 2005). Predator type influenced both aggressive and vigilance behavior, whereas shrub height independently affected only vigilance responses. In contrast, stationary observation and threatening behavior were unaffected by either predator identity or measured nest-site characteristics, which represents relatively invariant defensive tactics. Such context-dependent antipredator plasticity enables Asian tits to allocate energy efficiently under variable predation risk, that is critical for their reproductive success.

##  Supplemental Information

10.7717/peerj.21608/supp-1 Supplemental Information 1 Defensive behaviors of Asian tits in response to different predators and characteristics of nest sites

## Additional Information and Declarations

### Field Study Permissions

The following information was supplied relating to field study approvals (*i.e.*, approving body and any reference numbers): This field survey was approved by Zuojia Nature Reserve and Forestry Bureau of Jilin Province (Approval No.: (2006) 178).

### Data Availability

The following information was supplied regarding data availability: Raw data are available in the Supplemental File.
